# Oral Peanut Challenge Identifies an Allergy but the Peanut Allergen Threshold Sensitivity Is Not Reproducible

**DOI:** 10.1371/journal.pone.0053465

**Published:** 2013-01-09

**Authors:** Susanne Glaumann, Anna Nopp, S. G. O. Johansson, Magnus P. Borres, Caroline Nilsson

**Affiliations:** 1 Department of Clinical Science and Education, Södersjukhuset, Centre for Allergy Research, Karolinska Institutet, Stockholm, Sweden; 2 Sach’s Children’s Hospital, Södersjukhuset, Stockholm, Sweden; 3 Department of Medicine, Clinical Immunology and Allergy Unit, Karolinska Institutet, Stockholm, Sweden; 4 Thermo Fisher Scientific, Uppsala, Sweden; 5 Department of Pediatrics, Institute for Clinical Sciences, The Sahlgrenska Academy at the University of Gothenburg, Sweden; University of Southampton School of Medicine, United Kingdom

## Abstract

**Background:**

Double-blind placebo-controlled food challenge, DBPCFC, the gold standard for diagnosing food allergy, is time-consuming and potentially dangerous. A basophil allergen threshold sensitivity test, CD-sens, has shown promising results as a diagnostic tool in food allergy.

**Objectives:**

To evaluate the reproducibility of oral peanut challenge and compare the outcome to CD-sens in peanut-sensitized children.

**Methods:**

Twenty-seven children (4–19 years) underwent a DBPCFC followed by a single-blind oral food-challenge. The peanut challenges (1 mg to 5 g) were evaluated by severity scoring. Blood samples were drawn for CD-sens before the two first challenges.

**Results:**

Thirteen children (48%) did not react at any of the challenges. Fourteen reacted at both peanut challenges but not to placebo. Only two of these children reacted at the same threshold dose and with the same severity score. All other children scored differently or reacted at different doses. For children with a positive challenge the geometric mean of the ratio of the doses was 1.834 (p = 0.307) and the arithmetic mean of the difference between the severity scores was 0.143 (p = 0.952). No association was obtained between the two peanut challenges regarding severity score (r_s_ = 0.11, p = 0.71) or threshold dose (r_s_ = 0.35, p = 0.22). Among the children positive in peanut challenge, 12 were positive in CD-sens. Two were low-responders and could not be evaluated. Geometric mean of the ratio of CD-sens values in children with a positive challenge was 1.035 (p = 0.505) but unlike for the severity score and the threshold dose the association between the two CD-sens values was strong (r_s_ = 0.94, P<0.001).

**Conclusions:**

For a positive/negative test the reproducibility is 100% for both peanut challenge and CD-sens. However, a comparison of the degree of allergen threshold sensitivity between the two tests is not possible since the threshold dose and severity scoring is not reproducible.

## Introduction

Peanuts are one of the most common foods causing allergic reactions in children [1], [2]. The severity of the reactions vary but can be life threatening [3] and lead to a decreased quality of life [4], [5]. Peanut allergy is usually diagnosed by case history, skin prick test (SPT) and/or immunoglobulin E antibody (IgE-ab) determination. However, the diagnosis often needs to be confirmed by a food challenge. Double-blind placebo-controlled food challenge (DBPCFC) is considered the gold standard [6], [7] and is an attempt to mimic real life exposure under standardized conditions. In addition, DBPCFC is the accepted reference test for food allergy when new diagnostic methods or therapies are evaluated [8]. However, DBPCFC is time consuming and associated with risks of severe allergic reactions. It is also difficult to objectively evaluate if a DBPCFC is positive or negative or to determine the severity of the allergy [9]. Besides, placebo reactions may occur in up to 13% of the DBPCFC [10].

The reproducibility of the severity of the reactions and the threshold doses at DBPCFC has to our knowledge so far not been properly documented. A score system for low dose DBPCFC for peanut integrating both symptoms and dose (<100 mg peanut protein) has earlier been published but the peanut challenges were not repeated to evaluate the reproducibility [11]. In addition there is no worldwide accepted score system available to evaluate both the severity of the reactions and threshold doses making it hard to compare the results from different studies.

New promising diagnostic tools like basophil allergen threshold sensitivity tests have been evaluated in allergy [12], [13], [14], [15]. A basophil activation test represents the IgE-mediated inflammatory pathway of the allergic response [16], [17]. In IgE-mediated allergy tissue mast cells and basophils in the blood are sensitized with IgE-ab against the allergen to which the individual reacts. By stimulating the basophils in vitro with decreasing doses of the allergen, the smallest amount of allergen stimulating the cells can be detected by determination of CD63 up-regulation by flow cytometry. This is designated as the basophil allergen threshold sensitivity, CD-sens [18] and the method has been shown to be an alternative to complex allergen in-vivo challenges. A good correlation has been found between CD-sens and SPT-titration [12] and allergen challenge tests in allergic rhino conjunctivitis [12] and allergic asthma [15] suggesting that CD-sens is a relevant marker of in vivo allergen sensitivity.

The aim of this study was to evaluate the reproducibility of oral peanut challenges for the determination of the allergen threshold sensitivity and to compare it to CD-sens to peanut.

## Methods

### Study Population

This study includes 27 children who were part of a previous study (n = 38) where peanut allergy was confirmed by double-blind placebo-controlled food challenge (DBPCFC) [13]. The children, aged 4–19 years old, were referred to Sach’s Children’s Hospital, Stockholm for an oral peanut challenge. All children had a suspected peanut allergy, IgE-ab to peanut (≥0.35 kU_A_/L) in serum (ImmunoCAP®Thermo Fischer Scientific, Uppsala, Sweden) and/or a positive skin prick test (SPT) (≥3 mm) to peanut (Soluprick 1/20, ALK, Copenhagen, Denmark) at inclusion. Exclusion criteria were antihistamines or oral steroids taken 4 days prior to the challenge or a history of previous anaphylactic reactions to peanut. A DBPCFC followed by a single-blind oral food-challenge (SBOFC) was performed. The SBOFC was blinded for the child and the parents. In children reacting with severe allergic symptoms at the first peanut challenge the SBOFC was not performed due to ethical reasons. Thus, among 38 children 27 underwent two peanut and one placebo challenge and were included in the present study. All three challenges were planned to take place within one month. The median number of days between the two peanut challenges was 14 (range 7–126). For five children this interval was rather long due to common illness or for social reasons. Of these, three children were negative in both food challenges and CD-sens. For the other two the time between the challenges was 56 (patient J2) and 126 (patient J17) days, respectively.

### Ethics Methods

The study was approved by the local ethics committee (Dnr 2008-1001-31/2) and the parents provided written consent.

### Challenge Testing

The challenges were performed using a challenge medium with 11% peanut and 7% fat [19]. Increasing doses of peanut were given every 30 minutes in up to 5 steps from 1 mg to 5 g; dose 1 = 0.001 g, dose 2 = 0.01 g, dose 3 = 0.1 g, dose 4 = 1 g and dose 5 = 5 g. The smaller increase between dose 4 and 5 is based on the experiences from our routine DBPCFC because most children are unable to eat 91 g of the challenge medium, chocolate ball. A negative test was defined as no allergic symptoms during two hours after challenge completion. All children stayed at least 2 hours after the challenge and longer if needed. Symptoms were scored according to Astier et al. [20] (Table 1).

**Table 1:** Symptom score according to Astier [20] to evaluate clinical reactions in DBPCFC.

Symptom score	Symptoms
0	**no symptoms**
1	abdominal pain that resolved without medical treatment, rhino conjunctivitis or urticaria <10 paplers, rash
2	**one organ involved,** *abdominal pain requiring treatment, *generalized urticaria**,** *non laryngeal angioedema**,** *mild asthma (cough, fall ofpeak expiratory flow <20%)
3	**two organs involved** (of symptoms mentioned under 2)
4	**three organs involved** (of symptoms mentioned under 2**) or asthma** requiring treatment or laryngeal edema, or hypotension
5	**cardiac and respiratory symptoms requiring hospitalization in the intensive care unit**

### Blood Sampling

After preparation of the skin with local anaesthesia, a peripheral venous catheter was provided. Before the two first challenges a blood sample was drawn and stored at +4°C for a maximum of 24 hours for CD-sens analyses.

### Basophil Analyses

Basophil allergen threshold sensitivity, CD-sens [12], [18] was calculated by stimulating the cells with decreasing concentrations of peanut extract. The same batch of peanut raw material was used in all oral challenges and CD-sens analyses. The stock solution, 1/20 weight/volume, was further diluted 1/50 and designated 1 000 arbitrary units per mL (AU/mL). Each sample was tested with 0.1–1000 AU/mL. In summary, anti- FcεRI (Bühlmann Laboratories AG, Schönenbuch, Switzerland) and N-formyl-methionyl-leucyl-phenylalanin (fMLP) (Sigma Chemilcal Co, St. Louis, Missouri, US) were used as positive controls and RPMI as negative control. Leucocytes were stained with CD63 (Immunotech, Marseille, France) and CD203c (Immunotech) and counted in a Navios flow cytometer (Beckman Coulter, Inc., Fullerton, CA, USA). The cut-off determining a positive allergen test was set to 5% of CD63-positive basophils [12], [18].

Individuals with basophils, which after anti-FcεRI stimulation, i.e. the positive control, responded with 0–5% CD63-upregulation were regarded as non-responders. For individuals with a response between 5–16% (low-responders) the results should be interpreted with caution. The cut off 16% was calculated from the positive controls (mean 76%, −3SD) of an in-house reference material of 264 allergic children and adults [13].

Basophil allergen threshold sensitivity was measured as the lowest allergen concentration giving 50% (LC50) of maximum CD63 up regulation. CD-sens is defined as the inverted value for LC50 multiplied by 100 and describes the degree of the patient’s allergen threshold sensitivity, that is a positive relation between the CD-sens value and the degree of the patient’s allergen sensitivity. [18].

### Statistics

In patients reacting at both challenges, Wilcoxon Signed Rank Test was used to test differences between the first and the second challenge for Astier score, threshold dose and CD-sens. The values for threshold dose and CD-sens were tested on log-transformed values, thus testing the relative change. The differences between the first and the second challenge are presented in Bland-Altman plots and the associations between first and second value were assessed with Spearman rank order correlation (r_s_). No adjustment for multiple testing has been performed. Thus, significant results should be regarded as descriptive and explorative. Statistical analysis was carried out using IBM SPSS Statistic 20.0, Chicago, Ill, USA. A p-value <0.05 was considered significant.

## Results

### Patient Characteristics

The median age of the 27 children in the study was 12.2 years (range 5.4–19) and 48% were girls. All 27 children had a diagnosis of peanut allergy and where IgE-sensitized to peanut. Thirteen children had a clinical convincing history of peanut allergy and the other fourteen children were diagnosed with peanut allergy due to results of tests performed in routine clinical work-up.

### Oral Food Challenge

**Figure 1:**
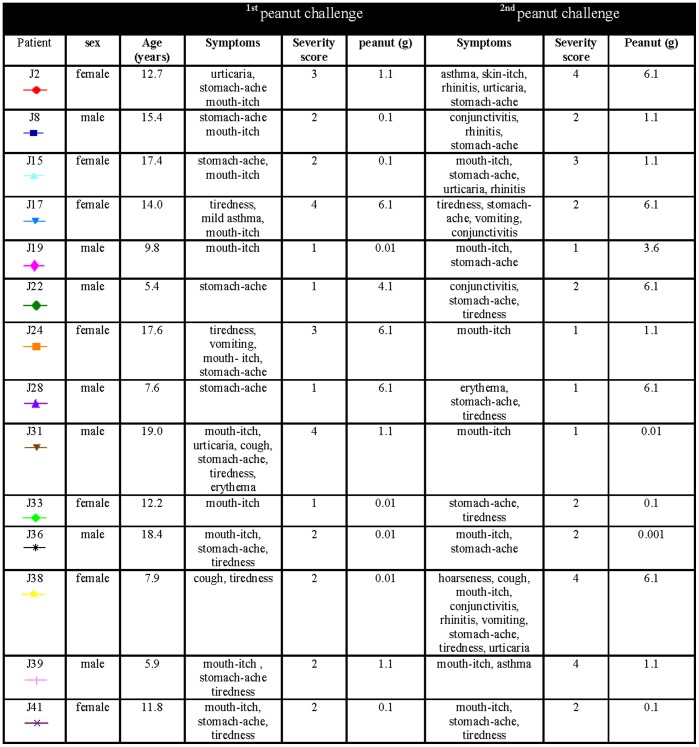
Age, sex, symptoms and Astier severity score [**20**] in children with two positive peanut challenges.

**Figure 2:**
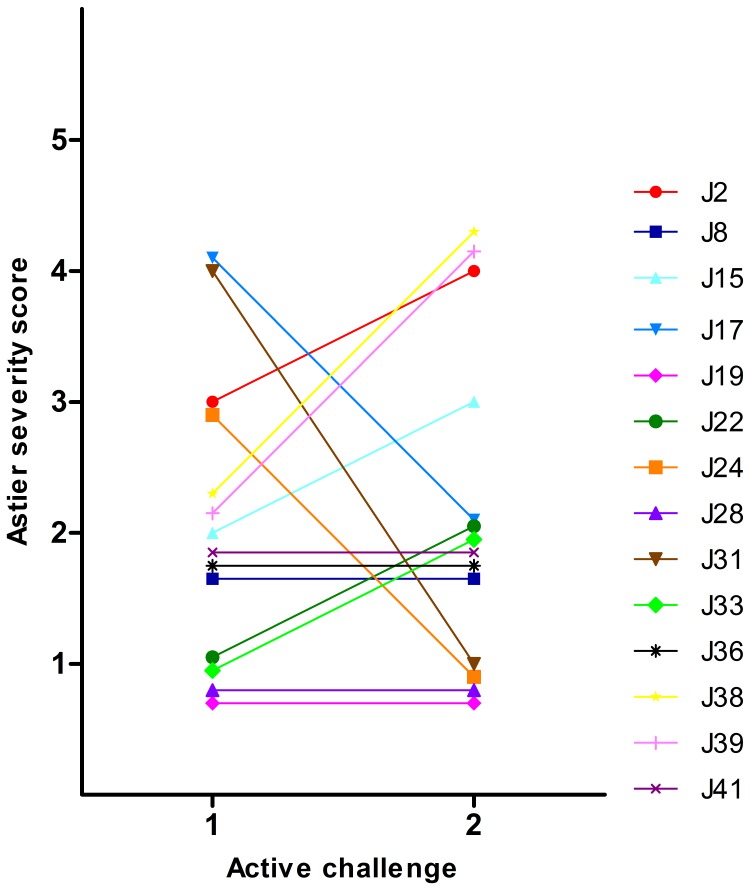
Severity scores according to Astier [**20**] in the same child at the two peanut challenges.

**Figure 3:**
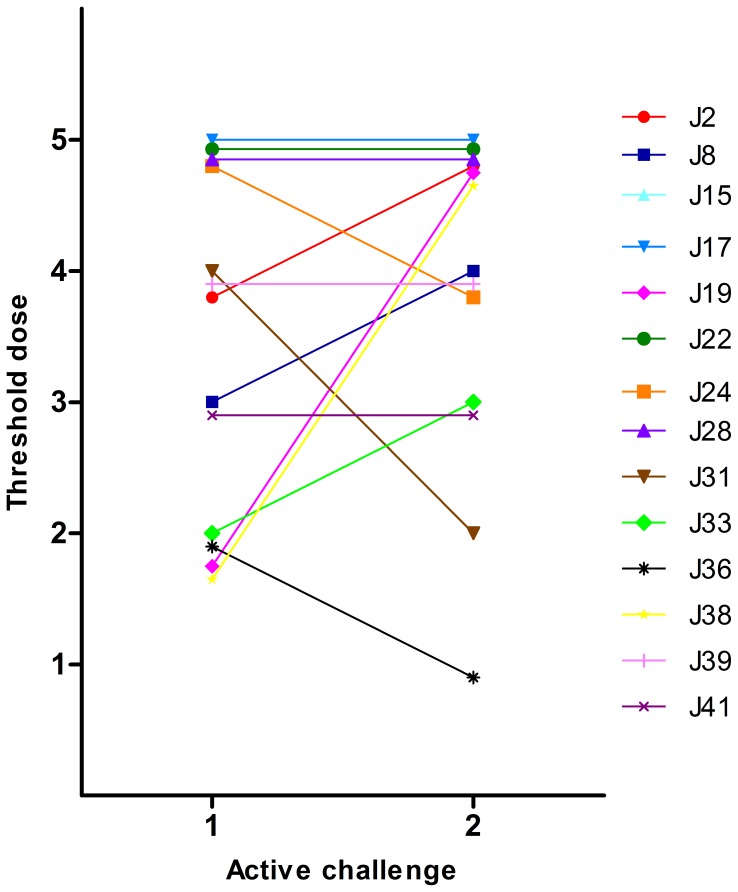
Threshold doses of peanut in the same child at the two peanut challenges. The amount peanut eaten were divided into 5 dose steps: Dose 1 = 0.001 g; Dose 2 = 0.01 g; Dose 3 = 0.1 g; Dose 4 = 1 g; Dose 5 = 3.6–5 g.

**Figure 4:**
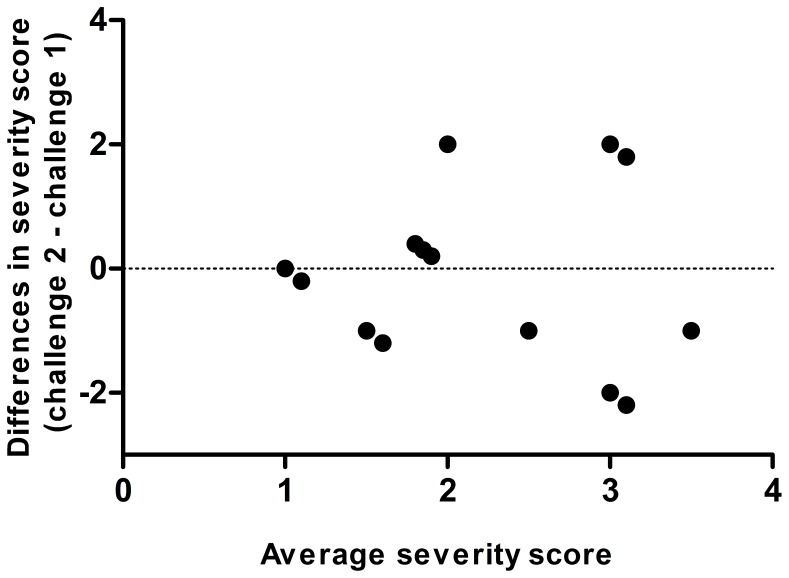
The differences in severity score (challenge 2–challenge 1) in each child with positive peanut challenges, presented as a Bland-Altman plot. The arithmetic mean of the difference between the severity scores was 0.143.

**Figure 5:**
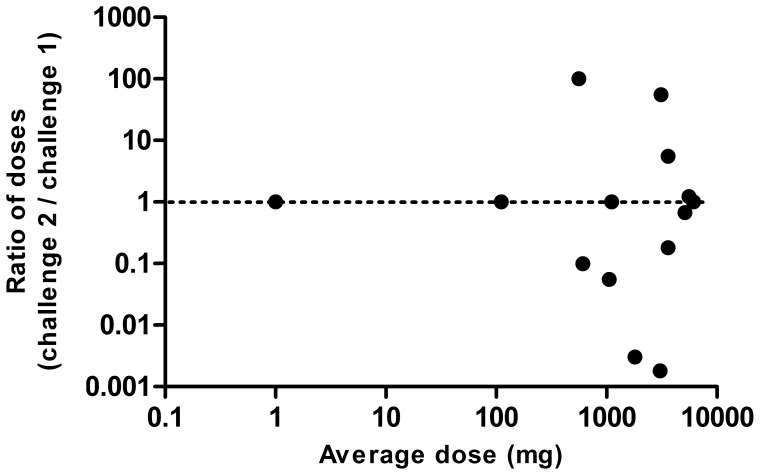
The differences in the ratio of the dose, mg (challenge 2/challenge 1) in each child with positive peanut challenges, presented as a Bland-Altman plot with logarithmically transformed data. The geometric mean of the ratio of the doses was 1.834.

The severity of the reactions was scored according to Astier et al. [20] (Table 1). Fourteen children (52%) reacted with allergic symptoms at both peanut challenges, but not on placebo, and were considered as positive (Figure 1). Two children (J28, J41) reacted at the same severity score and had the same threshold dose at the two peanut challenges while all other children (n = 12) scored differently or reacted at different threshold doses (Figure 2 and 3). Thirteen children (48%) did not react at any of the three challenges. All children negative in the challenges consumed full doses (6.1 g peanut). In children with a positive challenge the arithmetic mean of difference in the severity scores (challenge 2 - challenge 1) was 0.143 (p = 0.952) (Figure 4) and the geometric mean of the ratio of the doses (challenge 2/challenge 1) was 1.834 (p = 0.307) (Figure 5). No association was obtained between the first and the second peanut challenge regarding severity score (r_s_ = 0.11, p = 0.71) or threshold dose (r_s_ = 0.35, p = 0.22).

### CD-sens

**Figure 6:**
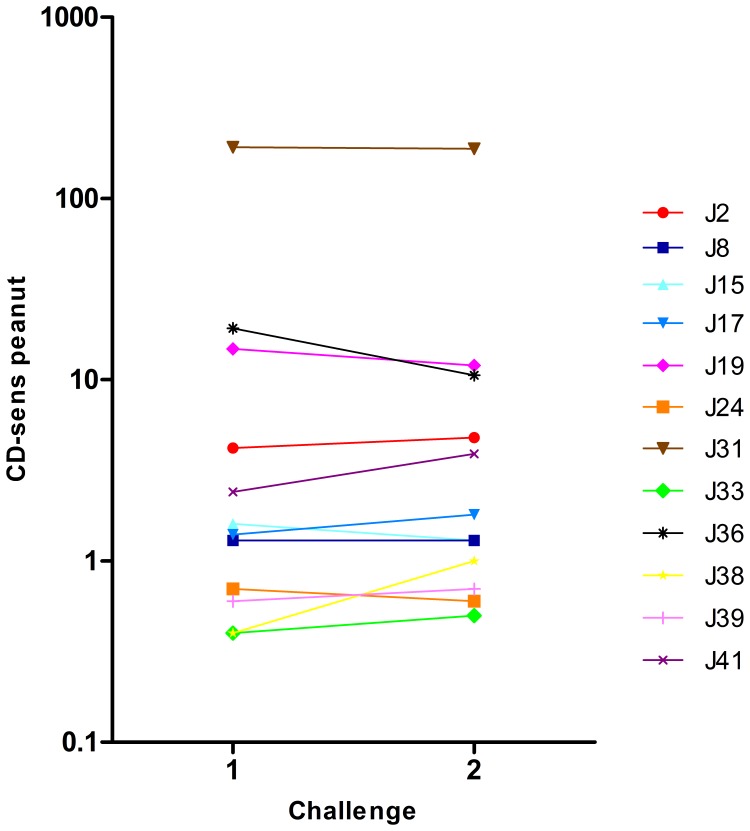
Peanut CD-sens values in the same child at the two peanut challenges.

**Figure 7:**
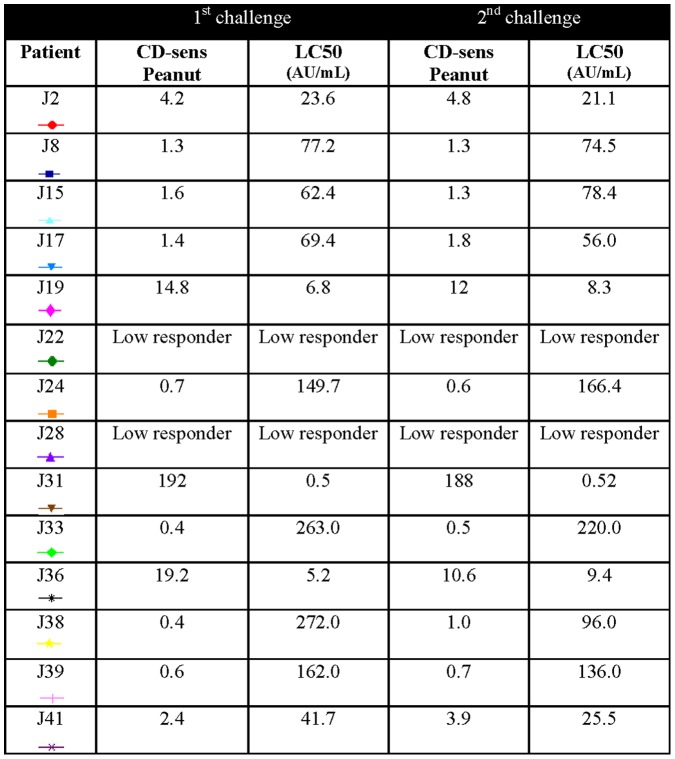
Peanut CD-sens values in children with two positive peanut challenges.

**Figure 8:**
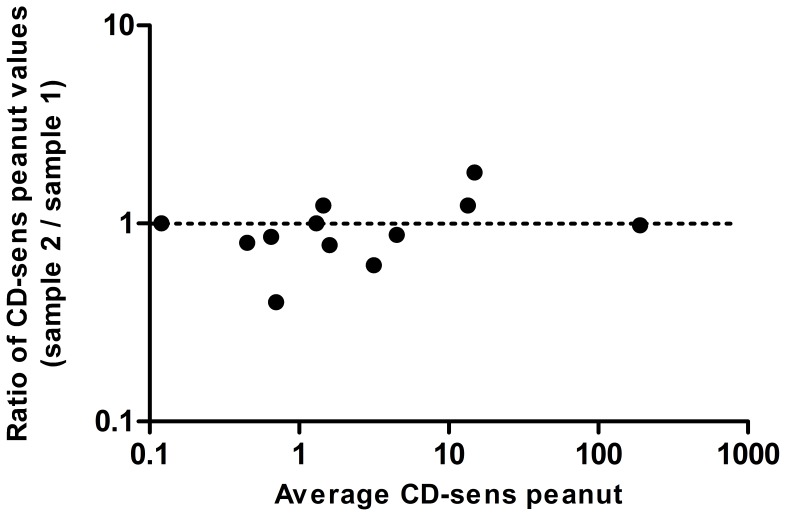
The differences in the ratio for peanut CD-sens values (challenge 2/challenge 1) in each child with positive peanut challenges, presented as a Bland-Altman plot with logarithmically transformed data. Geometric mean of the ratio of CD-sens values was 1.035.

CD-sens was performed on blood drawn before the first and second challenges in all children (n = 26) except one. In this child, blood was collected at the second and third challenges due to a misunderstanding. All children with a positive CD-sens at the first challenge where also CD-sens positive at the second challenge. The peanut CD-sens values are presented in figure 6 and 7. Twelve of 14 children with positive challenges were positive in CD-sens to peanut. The remaining two could not be evaluated since they were low responders i.e. had a too low response to the positive control anti-FcεRI (<16%). Three children had slightly positive values in CD-sens (0.3–0.5) but were negative in challenge. However, all 10 children negative in CD-sens were also negative at both peanut challenges. The geometric mean of the ratio of CD-sens values (challenge 2/challenge 1) in children with a positive challenge was 1.035 (p = 0.505) (Figure 8) and the association between the two CD-sens values was strong (r_s_ = 0.94, P<0.001).

## Discussion

The aim of this study was to evaluate the reproducibility of oral peanut challenges (DBPCFC and SBOFC) for the determination of the allergen threshold sensitivity and to compare it to CD-sens to peanut. However, in contrast to CD-sens it was not possible to quantitatively estimate the allergen threshold sensitivity with oral peanut challenge in a reproducible way. Thus, a comparison of the degree of allergen threshold sensitivity obtained by oral peanut challenge with CD-sens, was not possible to achieve.

The DBPCFC is an attempt to mimic real life exposure under standardized conditions and is today the gold standard for the diagnosis of food allergy [21], [22]. However, it is difficult to evaluate the reactions at DBPCFC [9] and each research group is using its own severity scores [11], [23], [24]. Besides, there are several factors affecting the outcome of a food challenge in addition to the amount of allergen given during challenge, for example aversion to the tested food and the evaluation of subjective symptoms [9]. Thus, reactions to placebo at the DBPCFC have been reported in a significant number [10]. This is in contrast to our study where no positive reactions to placebo occurred.

In the present study all children but two reacted with a different severity score or at another dose when the peanut challenge was repeated. Several studies have tried to determine the threshold dose in peanut allergic patients to predict the clinical sensitivity [24], [25], [26]. Van der Zee and colleagues [24] aimed to evaluate if the presence of risk factors for a severe allergic reaction to peanut is associated with the patient’s clinical sensitivity by determine the eliciting dose of peanut in a DBPCFC and thereby predicting the severity of the allergic reaction. However, they were not able to find a relation between the threshold dose in DBPCFC and the severity of a previous reaction at home. Besides, Skirpak and colleagues [27] evaluated a 23 weeks oral immunotherapy to milk with DBPCFC before and after treatment. The outcome of the food challenge was based on the lowest dose causing a reaction and not from changes in the severity of the reactions. In that study 29% of the patients in the placebo group (n = 7) did not react at the same dose at the second DBPCFC. Although the numbers of patients in that study was low, the results indicate that food challenges can be difficult to reproduce [27]. These findings are in line with our observations showing that the severity of the clinical reaction of an oral peanut challenge is not reproducible.

A food challenge can tell you if a patient is allergic or not [13] but when we in this paper investigated the allergen threshold sensitivity and its reproducibility we could not demonstrate a reproducibility of the peanut challenges for determination of allergen sensitivity.

In CD-sens the correlation between the two CD-sens values was strong. A good correlation does not always mean that there is a high reproducibility, but with a high reproducibility there should be a good correlation. For the peanut challenge we could not demonstrate any statistical differences or correlations for doses or symptoms between the two challenges. Thus the low correlation support that the reproducibility was poor for dose and severity score. Difficulties to determine allergen thresholds doses at food challenges have also been noted by others [28]. This is in contrast to CD-sens where we did not find any significant differences but a significant and strong correlation between the two CD-sens occasions. This is supporting that CD-sens measured at different time-points strongly are associated.

Since the CD-sens value is directly related to the allergen concentration of the preparation used and since allergen extracts are not satisfactorily standardized the CD-sens values cannot be compared between different allergens [15]. In this study we used, for the CD-sens analyses, an extract of the same raw material used at the oral challenges. However, since we were not able to determine the patient’s allergen threshold sensitivity by oral challenge we could not compare the severity of the peanut challenge reaction with CD-sens to peanut.

CD-sens has previously been shown to have a good correlation with clinical tests of allergen sensitivity e.g. SPT-titration [12] and allergen challenge in rhino-conjunctivitis [12] and allergic asthma [15]. Interestingly, in allergic asthma there was an excellent correlation between CD-sens and the allergic inflammation behind the bronchial sensitivity but not between CD-sens and the non-specific bronchial hyper-reactivity [12]. Thus, we propose that CD-sens can be used in food allergy to predict the patient’s basic allergen sensitivity. CD-sens allows evaluation and monitoring over time of the degree of allergen sensitivity with no risk for the patient. In addition, CD-sens is also, cost effective and time saving.

The strength of the present study is that all oral peanut challenges were performed in the same clinic with the same experienced senior physician grading the patient’s clinical reaction and symptom score. An error introduced by performing the second peanut challenge as SBOFC, e.g. that the nurse performing the test was aware of the results of the first challenge, would have resulted in a falsely too good correlation. However, no correlation was found.

Since this is a rather small study the results should be interpreted with caution. Another limitation is that all children with severe anaphylactic reaction at the first peanut challenge were excluded for clinical and ethical reasons when the study was planned, we expected a severe reaction to re-occur. It should also be noted that the blood samples for CD-sens analyses were drawn at the first two challenges and not at the two peanut challenges.

To our knowledge there is no published study investigating the reproducibility of oral food challenge regarding both severity score and dose. Thus, clinical trials using DBPCFC for testing the outcomes of treatments [24], [27] can only be correctly evaluated if the study includes information about the reproducibility of the challenges.

We conclude that we were not able to determine the severity of an allergic reaction to peanut since the children reacted at a different dose or with another severity score when the challenge was repeated. However, for a positive/negative test result, the concordance between the two CD-sens values and the two peanut challenges were 100% respectively, but comparison of the degree of allergen threshold sensitivity between the two tests was not possible to achieve since the threshold dose and severity scoring were not reproducible.

If DBPCFC should continue to be used as gold standard for the diagnosis of a food allergy and to predict the severity of a potential clinical reaction, its precision and reproducibility must be documented and improved. Besides, it is of great importance to develop a scoring system integrating both the symptom severity grade and the allergen dose and which can be used world-wide. CD-sens shows promising result with only small variations between the two test occasions but further studies of the role of CD-sens in food allergy diagnosis and treatment trials are most urgent.
